# A critical analysis of the quadripartite one health joint plan of action

**DOI:** 10.1016/j.onehlt.2026.101597

**Published:** 2026-09-27

**Authors:** Júlio Belo Fernandes

**Affiliations:** Egas Moniz Center for Interdisciplinary Research (CiiEM), Egas Moniz School of Health & Science, Caparica, 2829-511 Almada, Portugal; Nurs* Lab, Caparica, 2829-511 Almada, Portugal

**Keywords:** One health, Health policy, Global health governance, Integrated surveillance

## Abstract

The Quadripartite One Health Joint Plan of Action (2022–2026), now extended to 2029, represents an important effort to consolidate One Health as a global policy framework. This article critically examines the Plan's achievements and structural constraints. Key strengths include the consolidation of political commitment, the formalisation of institutional alignment at global level, and the prioritisation of prevention, preparedness, and integrated surveillance. Environmental governance was formally included, recognising ecosystem degradation and biodiversity loss as upstream drivers of risk. However, although later guidance added an operational layer, the Plan remains predominantly normative, its financing guidance says more about mobilising investment than about sustaining recurrent functions, and participatory mechanisms remain predominantly consultative. Its framing continues to privilege health security over long-term socioecological determinants and routine practice. The article proposes priorities for the structured review and next phase: clearer implementation pathways with comparable reporting, coordinated investment, participatory governance, and One Health embedded in routine practice.

## 1 Introduction

The Quadripartite One Health Joint Plan of Action (2022–2026) represents a landmark effort to consolidate One Health as a shared global policy framework [1]. Developed in the aftermath of the COVID-19 pandemic, which exposed the costs of fragmented cross-sectoral preparedness and accelerated calls for coordinated prevention at the human–animal–environment interface, the plan was not intended as an implementation blueprint, but rather as a strategic alignment instrument designed to translate long-standing conceptual consensus on the interdependence of human, animal, and environmental health into coordinated global direction. The plan is explicit about this function: it presents itself not as a binding policy instrument but as a framework for action, setting out activities the four organisations can offer jointly in support of countries, and as a living document open to adjustment [1]. In August 2026 the Quadripartite extended its implementation period to 2029 and announced a structured review to take stock of implementation, document lessons learned, and identify adjustments for the next phase [2]. That review makes a critical assessment timely, one that evaluates the plan against its intended strategic function and identifies the structural gaps that should inform its next phase.

One of the plan's principal strengths lies in its political and symbolic significance [1]. By formalising collaboration among four major international organisations, the Joint Plan of Action elevated One Health from a largely aspirational discourse to a recognised global policy priority [3]. In this sense, the plan formalised institutional alignment at the global level and strengthened the legitimacy of One Health as a guiding paradigm for addressing complex, transboundary health challenges.

The plan also made a substantive contribution by prioritising prevention, preparedness, and integrated surveillance across human, animal, and environmental systems. Its emphasis on zoonotic diseases, antimicrobial resistance, and emerging threats reflected the systemic failures revealed by recent global crises [4]. Notably, the formal inclusion of the United Nations Environment Programme marked a significant shift toward recognising ecological degradation, biodiversity loss, land-use change, and ecosystem disruption as upstream drivers of health risks across species, rather than as contextual or secondary concerns.

However, when assessed against its ambition to “operationalise” One Health, the Joint Plan of Action reveals structural limitations. Following Milazzo et al. [5], operationalisation refers to translating integrated human, animal, and environmental health into practical strategies, policies, and actions; the analysis here focuses on the institutional arrangements, responsibilities, resources, and coordination mechanisms through which that translation is sustained, whereas normative guidance states what ought to be done without specifying them. Although later guidance has added an operational layer, the plan itself remains predominantly normative, articulating strategic orientations without consistently specifying the institutional conditions, accountability mechanisms, and resources through which integrated action is to be sustained across governance levels and sectors. The limitation is not confined to human health systems: responsibilities are equally fragmented across animal health services, environmental monitoring, and ecosystem management [6]. In 2023 the Quadripartite published an operational addendum, a guide to implementing the plan at national level, which sets out three pathways and five steps and recommends a multisectoral coordination mechanism at an appropriate political level, supported by a secretariat, technical working groups, and a sufficient budget [7]. A translation architecture therefore exists. What remains under-specified is its binding force: uptake is encouraged rather than expected, Quadripartite support is repeatedly qualified as subject to available resources, and reporting back to the global level is voluntary, with countries invited to prepare annual reports and, ideally, to share them [7]. Consistency, accountability, and resourcing thus depend on conditions the plan does not address. Experience that predates the plan shows why these conditions matter. The scoping review by Milazzo et al., covering One Health programmes reported up to mid-2023, describes capacity in Switzerland as supported by a detailed roadmap and a legal body for coordination, implementation in Ethiopia as slow and incomplete, with formal linkages still absent, and integration in Kenya as strong at national level but weaker subnationally [5].

This gap becomes particularly visible in relation to financing and resource mobilisation. Guidance here is not absent: the implementation guide asks countries to cost their plans, map the investment landscape, involve ministries of finance, and treat a coordinated multisectoral budget as a way of avoiding competition between sectors [7], while the Quadripartite Joint Offer seeks catalytic funding for at least ten countries over three to five years so that they can demonstrate measurable impact and leverage further investment [8]. Both instruments nonetheless say more about mobilising and coordinating investment than about how recurrent cross-sectoral functions are embedded and sustained within ordinary public budget processes once catalytic support ends, a concern the Quadripartite itself signals in noting that the newer global funds focus largely on preparedness and response [8]. Greater specificity is possible: Thailand's National Action Plan for Health Security for 2023 to 2027 proposes piloting a shared budgeting system for cross-sectoral activities, including One Health [9]. Whether such arrangements deliver recurrent financing remains to be shown. Meanwhile, the plan's own theory of change assumes that political will and financing are in place, or can be mobilised, at global, national, and local levels [1], so the durability of integrated One Health functions depends on circumstance rather than design.

Governance arrangements represent another area of tension. The plan emphasises, in principle, multisectoral collaboration and community engagement, yet decision-making remains largely centralised within international institutions and national authorities. Participation of local communities, frontline professionals, and holders of context-specific or indigenous knowledge is predominantly consultative rather than co-creative. Here, co-creation refers to meaningful joint involvement in defining problems, setting priorities, designing responses, and evaluating them, with traceable influence over decisions rather than consultation alone. The One Health High-Level Expert Panel, which advises the Quadripartite, now recommends participatory governance and co-ownership, with communities meaningfully engaged in the design, implementation, and monitoring of One Health actions [10]. The mechanisms set out for national implementation, by contrast, are stakeholder mapping, consultations, and validation workshops, with financial and resource decisions remaining with national authorities [7]. In the scoping review cited above, 8 of 77 programmes (10.4%) involved local communities or Indigenous groups [5]. The plan itself gives local stakeholders a central role in identifying local challenges and in designing locally adapted solutions, and its theory of change lists insufficient community inclusion among the barriers to be removed [1]. The gap therefore lies between the plan's own principle and the mechanisms offered to enact it.

The substantive framing of the plan further narrows its scope by privileging a health security perspective centred on infectious threats and emergency preparedness. The plan is not silent on ecological ambition: it adopts a definition of One Health that aims to balance and optimise the health of humans, animals, plants, and ecosystems, and its objectives include protecting and restoring biodiversity and preventing ecosystem degradation [1]. The difficulty lies in the passage from principle to implementation, where the environmental dimension in particular tends to be addressed as a source of risk to human health rather than as a domain with intrinsic health, resilience, and sustainability goals [11]. The baseline the plan inherited was already asymmetrical: in the scoping review cited above, 42 of the 77 programmes, most of them predating the plan, involved only the human and animal sectors, none addressed human–environment or animal–environment relations alone, and the action concerned with protecting and restoring ecosystems was pursued in 5, the lowest proportion recorded in that analysis [5]. Four years on, the same expert panel describes environmental integration as a systemic gap in which environmental stakeholders have remained under-represented or marginalised [10]. Broader determinants, such as intensive farming, habitat fragmentation, pollution, and climate-related ecosystem change, receive less attention as ongoing drivers of vulnerability. This imbalance risks reducing One Health to a reactive risk-management tool rather than consolidating it as a genuinely integrative socioecological paradigm.

Relatedly, the plan places limited emphasis on everyday systems where One Health is enacted over time, including primary animal health services, environmental stewardship, food system governance, and community-based practices at the human–animal–environment interface. Many contemporary challenges, such as antimicrobial resistance, food-borne disease, and environmentally mediated non-communicable conditions, emerge through chronic, cumulative interactions within these systems rather than through discrete crisis events [12], [13], [14]. Without stronger integration of these domains, One Health remains policy-facing but insufficiently grounded in routine practice across sectors.

Taken together, these limitations delimit rather than negate the value of the plan, which is best understood as a foundational alignment exercise rather than a definitive operational solution. Its principal weaknesses stem from translation mechanisms that remain non-binding and unevenly resourced, a security-dominated framing, and insufficient attention to the sustained governance of interconnected socioecological systems.

## 2 Toward a renewed One Health Joint Plan of Action: principles for greater clarity and feasibility in the next phase

The structured review offers an opportunity to connect global alignment more clearly to feasible implementation, while respecting the diversity of governance contexts in which One Health must operate [2].

First, a renewed plan should be more explicit about what countries are expected to put in place and how progress is to be known. The implementation guide already describes how coordination can be organised; what remains to be specified is the minimum set of functions that national and subnational systems are expected to perform across human health services, animal health systems, food and agriculture governance, and environmental management, while leaving open the structures through which each country performs them. Since uptake and reporting are voluntary, it also means stating what countries are expected to report on, in a form comparable between them; voluntary evaluation approaches already in use, such as the Joint External Evaluation under the International Health Regulations and the Performance of Veterinary Services Pathway, offer a basis [10]. It also means stating which elements of Quadripartite support countries can rely on, rather than support offered only as resources allow.

Second, the plan should be more explicit in how it frames financing. Greater clarity is needed on how countries can align resources across sectors to sustain integrated functions over time, how co-benefits across human health, animal health, food systems, and environmental protection are recognised in investment decisions, and how catalytic or project-based support is expected to convert into recurrent financing once it ends. Without such clarity, integration risks remain rhetorical while financing continues to reinforce sectoral silos.

Third, a renewed plan should be more explicit in its approach to governance and participation. Multisectoral collaboration and community engagement should be framed not only as guiding values but as governance design requirements with clear operational consequences. This means specifying how communities, frontline professionals, environmental practitioners, and holders of context-specific and indigenous ecological knowledge contribute to agenda-setting, risk assessment, and priority-setting in ways that are genuinely influential rather than merely consultative, and how participation is balanced with decision-making authority and accountability. Co-management arrangements with a statutory basis, such as those governing wildlife research and management in Inuit Nunangat, where communities help frame the questions and share in the decisions from the outset, offer principles from which such mechanisms could be derived [15].

Fourth, the plan should be more explicit in balancing health security priorities with the broader socioecological determinants of health across species. A renewed framework should clarify how ongoing drivers such as land-use change, biodiversity loss, intensification of animal production, pollution, and climate-related ecosystem disruption are to be addressed as persistent sources of risk and vulnerability. This would reposition animals and ecosystems not merely as reservoirs or pathways of human disease, but as domains with intrinsic objectives. The plan already envisages a One Health indicator framework that includes the health of the environment [1]; the structured review could establish whether it has been produced and whether ecosystem health is counted in it as an outcome rather than as context.

Finally, the plan should be more explicit in recognising the role of everyday systems and routine practice in sustaining One Health. To anchor One Health in continuous practice rather than episodic response, the minimum set of functions proposed above could include, in every national system and whoever performs them, standing coordination that meets and decides between emergencies; routine data exchange between human health, animal health, and environmental services; joint risk assessment applied to chronic as well as acute threats; antimicrobial stewardship across sectors; and a defined route through which a local signal reaches the level able to act on it. Attaching such functions to existing mandates, job descriptions, and budgets, rather than to new structures, would allow countries to organise them differently while making it possible to establish whether they are performed at all. This is consistent with the expert panel's recommendation to embed One Health competencies in sectoral training and professional job descriptions, and complements its call for formal subnational coordination systems by specifying what must be done rather than which bodies should do it [10].

Decisions on the plan itself rest with the Quadripartite, which extended the implementation period in August 2026, before the congress [2]. At the ninth World One Health Congress, held in Lisbon in September 2026, the Quadripartite partners emphasised implementation, science, and investment, representatives of Malawi, Singapore, and Uzbekistan described their use of the plan and its implementation guide [16], and the expert panel presented the recommendations cited above, including the transformation of the plan from a technical document into a measurable, operational system [10]. Several of the gaps identified in this analysis are thus named by the panel itself as barriers to implementation, four years into the plan.

## 3 Conclusion

The Quadripartite One Health Joint Plan of Action represents a critical step in consolidating One Health as a global policy paradigm. Its value lies primarily in political alignment and institutional legitimacy. The extended implementation period, and the structured review that accompanies it, offer an opportunity to move from alignment to grounded action, strengthening implementation pathways, governance design, and substantive scope while preserving the integrative vision that underpins One Health.

## CRediT authorship contribution statement

**Júlio Belo Fernandes:** Writing – review & editing, Writing – original draft, Conceptualization.

## Consent for publication

Not applicable.

## Ethics approval and consent to participate

Not applicable.

## Funding

This work was supported by FCT - Fundação para a Ciência e Tecnologia, I.P. by project reference UID/4585/2025 and DOI identifier https://doi.org/10.54499/UID/04585/2025

## Declaration of competing interest

The authors declares that there are no conflicts of interest regarding the publication of this paper.

## Data Availability

No data was used for the research described in the article.

## References

[1] Food and Agriculture Organization of the United Nations (FAO), United Nations Environment Programme (UNEP), World Health Organization (WHO), World Organisation for Animal Health (WOAH) (2022). One Health Joint Plan of Action (2022–2026). https://www.woah.org/app/uploads/2022/04/one-health-joint-plan-of-action-final.pdf.

[2] Food and Agriculture Organization of the United Nations (FAO), United Nations Environment Programme (UNEP), World Health Organization (WHO), World Organisation for Animal Health (WOAH) (2026). Quadripartite Collaboration Extends the One Health Joint Plan of Action to 2029. https://www.woah.org/en/quadripartite-collaboration-extends-the-one-health-joint-plan-of-action-to-2029/.

[3] One Health High-Level Expert Panel (2023). One health action for health security and equity. Lancet.

[4] Astbury C.C., Demeshko A., Aguilar R., Mapatano M.A., Li A., Togño K.C. (2025). Wildlife policy, the food system and one health: a complex systems analysis of unintended consequences for the prevention of emerging zoonoses in China, the Democratic Republic of the Congo and the Philippines. BMJ Glob. Health.

[5] Milazzo A., Liu J., Multani P., Steele S., Hoon E., Chaber A.-L. (2025). One health implementation: a systematic scoping review using the quadripartite one health joint plan of action. One Health.

[6] Ribeiro C.D.S., van de Burgwal L.H.M., Regeer B.J. (2019). Overcoming challenges for designing and implementing the one health approach: a systematic review of the literature. One Health.

[7] World Health Organization (WHO), Food and agriculture Organization of the United Nations (FAO), United Nations Environment Programme (UNEP), World Organisation for Animal Health (WOAH) (2023). A Guide to Implementing the One Health Joint Plan of Action at National Level, Geneva. https://www.woah.org/app/uploads/2023/12/guide-to-implement-the-oh-jpa-v19-web.pdf.

[8] World Organisation for Animal Health (WOAH), Food and Agriculture Organization of the United Nations (FAO), United Nations Environment Programme (UNEP), World Health Organization (WHO) (2025).

[9] Ministry of Public Health of Thailand (2024). https://www.who.int/publications/m/item/thailand--national-action-plan-for-health-security-(naphs).

[10] One Health High-Level Expert Panel (OHHLEP) (2026). https://www.who.int/publications/m/item/enablers-and-barriers-to-implementing-the-quadripartite-one-health-joint-plan-of-action.

[11] Elnaiem A., Mohamed-Ahmed O., Zumla A., Mecaskey J., Charron N., Abakar M.F. (2023). Global and regional governance of one health and implications for global health security. Lancet.

[12] Winkler A.S., Brux C.M., Carabin H., das Neves C.G., Häsler B., Zinsstag J. (2025). The lancet one health commission: harnessing our interconnectedness for equitable, sustainable, and healthy socioecological systems. Lancet (London, England).

[13] World Health Organization (WHO) (2023). https://www.who.int/news-room/questions-and-answers/item/one-health.

[14] Food and Agriculture Organization of the United Nations, World Organisation for Animal Health, World Health Organization (2022).

[15] Sudlovenick E., Jenkins E., Loseto L. (2024). Comparative review of one health and indigenous approaches to wildlife research in Inuit Nunangat. One Health.

[16] World Health Organization (WHO) (2026). Advancing One Health Implementation and Investment: From Country Action to Sustainable Solutions. https://www.who.int/news/item/22-09-2026-advancing-one-health-implementation-and-investment--from-country-action-to-sustainable-solutions.

